# Vaccination strategies when vaccines are scarce: on conflicts between reducing the burden and avoiding the evolution of escape mutants

**DOI:** 10.1098/rsif.2022.0045

**Published:** 2022-06-29

**Authors:** Félix Geoffroy, Arne Traulsen, Hildegard Uecker

**Affiliations:** Department of Evolutionary Theory, Max Planck Institute for Evolutionary Biology, August-Thienemann-Strasse 2, 24306 Plön, Germany

**Keywords:** SIR model, vaccination strategies, vaccine escape

## Abstract

When vaccine supply is limited but population immunization urgent, the allocation of the available doses needs to be carefully considered. One aspect of dose allocation is the time interval between the first and the second injections in two-dose vaccines. By stretching this interval, more individuals can be vaccinated with the first dose more quickly, which can be beneficial in reducing case numbers, provided a single dose is sufficiently effective. On the other hand, there has been concern that intermediate levels of immunity in partially vaccinated individuals may favour the evolution of vaccine escape mutants. In that case, a large fraction of half-vaccinated individuals would pose a risk—but only if they encounter the virus. This raises the question whether there is a conflict between reducing the burden and the risk of vaccine escape evolution or not. We develop an SIR-type model to assess the population-level effects of the timing of the second dose. Trade-offs can occur both if vaccine escape evolution is more likely or if it is less likely in half-vaccinated than in unvaccinated individuals. Their presence or absence depends on the efficacies for susceptibility and transmissibility elicited by a single dose.

## 1. Introduction

Many vaccines are administered in two doses with a certain time interval between them. The second shot increases the strength and duration of protection. However, the first shot on its own already establishes some immunity. At the beginning of a vaccination campaign in a pandemic—such as in the current COVID pandemic—when population immunization is urgent but vaccine doses are scarce, the question arises whether the second shot should be delayed at the benefit of administering the first vaccine dose to more people more quickly. Even if half-vaccinated individuals are only partially immune, the overall reduction in infections may be greater in a population in which many people have some immunity than in a population in which fewer individuals have stronger immunity. In the current COVID pandemic, such a delay strategy has been adopted by the UK [1], while several other countries such as the USA stick to the interval between injections that has been applied in the original clinical trials and is therefore recommended by the manufacturer [2]. In early January 2021, the World Health Organization recommended to stretch the dosing interval of the first approved vaccine (Pfizer–BioNTech COVID-19 vaccine) from 21–28 days to 42 days in countries ‘experiencing exceptional epidemiological circumstances’ [3], which has for example been adopted by Germany, especially from April 2021 on [4]. Already preceding the current pandemic, mathematical models have compared the effects of a delay strategy in its extreme form—a one-dose strategy—and a two-dose strategy for cholera and influenza epidemics/pandemics [5,6]. Sparked by the COVID crisis, a series of models have been set up to assess when stretching the period between the two shots reduces the total number of SARS-CoV-2 infections [7–11].

However, there is another dimension to the problem, since the vaccination strategy does not only affect the dynamics of the current strain of virus, but may also influence the evolutionary dynamics of the virus (or another pathogen in other circumstances). This especially concerns the evolution of vaccine escape mutants against which the vaccine has no or reduced efficacy [11]. Vaccine resistance is generally rare [12]. Yet, the large case numbers in the current pandemic give the virus a lot of opportunity to replicate, mutate and adapt. It has been hypothesized that vaccine escape mutants evolve most easily in people who have only received one vaccine shot [13]. The reasoning behind this hypothesis is the following (similar to [14]). After the first dose, vaccinees have intermediate levels of antibodies [11,15]. This level is not enough to keep the viral load following exposure to the virus sufficiently low to avoid the occurrence of a large number of mutations, including vaccine escape mutations. At the same time, it gives a large advantage to mutant virus particles to which the antibodies bind only weakly. By contrast, in fully vaccinated individuals, the viral load is kept low such that mutations are unlikely to occur. In unvaccinated patients, the virus can initially replicate well and attain high numbers. However, the immune response is broader than the one elicited by the vaccine such that vaccine escape mutants do not have a great advantage over other viral genotypes. Hence, according to this reasoning, the evolution of vaccine escape is most likely at intermediate levels of antibodies, which are typical for half-vaccinated individuals. This raises the concern that the large number of half-vaccinated individuals in the delay strategy may drive the evolution of vaccine escape. Important counter-arguments to this reasoning bring forward (i) the small number of virus replications between infection and transmission in acute infections, which gives little time for mutations to appear and selection to act, and (ii) residual immunity that limits the growth of escape mutants if they appear [16,17].

Viral evolution can only occur in infected individuals. Thus, even if the above reasoning holds, whether vaccine escape evolves in a host population does not only depend on the number of partially immune individuals, but also on the number of infected individuals. If the delay strategy reduces the disease prevalence in the population, this may offset the increased probability of vaccine escape evolution within any one half-vaccinated patient and may actually decrease rather than increase the risk of vaccine escape (see the discussion in [16]). Hence, in the interplay of all effects, is there a trade-off between reducing the cumulative number of infections in the pandemic and minimizing the risk of escape mutants or not?

What we were missing in the current public debate is a quantitative epidemic model that includes the emergence of escape mutations and quantifies how the strengths of the various population-level effects compare to each other. We therefore set up an SIR-type model to dissect and quantify the considerations and verbal arguments outlined above. We chose a minimal model that is stripped down to the most essential components needed to study both aspects of the problem of dose allocation. This, of course, ignores much of the biological complexity and does not allow one to make immediate recommendations for vaccine strategies in the current pandemic. However, the transparency of the model makes it possible to develop a better intuition for the conditions under which there is a trade-off and those under which the same allocation strategy is optimal in both respects. We therefore hope that it can contribute to a better-informed discussion.

## 2. The model

**Figure 1.**
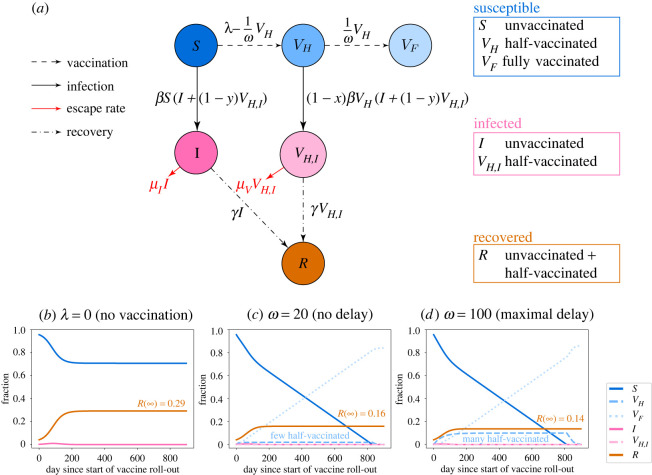
Model for the disease dynamics with a two-dose vaccine. (*a*) Flow diagram of the model defined by equation (2.1). The model corresponds to an extended SIR model, in which individuals can either be unvaccinated, vaccinated with the first dose only, or vaccinated with both doses. The first dose has an efficacy on susceptibility *x* and on transmissibility *y*. For simplicity, we assume that vaccination with both doses provides perfect protection from the virus. Since vaccine supply is limited, there are different strategies: either administering the second shots after the minimally required time interval between the two doses *ω*_min_ or delaying the second injection and giving the first dose to more people more quickly (*ω* > *ω*_min_). The model describes the dynamics in the absence of vaccine escape mutants. The rate at which such mutants emerge can be obtained from the number of infected individuals, where vaccine escape may possibly evolve at different *per capita* rates in unvaccinated and half-vaccinated patients. The allocation strategy affects the total number of individuals that become infected throughout the pandemic, but also the risk of vaccine escape evolution across the population. It is *a priori* not clear whether the same strategy minimizes both quantitites. (*b*) Example dynamics without vaccination. (*c*,*d*) Example dynamics of infection and vaccination with and without a delay in the second dose. In (*c*), the second dose is administered as soon as possible (*ω* = *ω*_min_). In this case, the fraction of half-vaccinated individuals is always low. In (*d*), the second dose is delayed (*ω* = *ω*_max_), leading to a much higher fraction of half-vaccinated individuals over time. The vaccination campaign starts during the pandemic, when a considerable fraction of the population has already been affected by the virus (*I*(0) = 2.4 × 10^−3^ and *R*(0) = 4.2 × 10^−2^). For illustrative purposes, we show the dynamics for maximal efficacies of the first dose *x* = *y* = 1.

We consider an SIR-type model, where individuals are either unvaccinated, vaccinated with the first dose only, or fully vaccinated with both doses. Since we are interested in the rate of de novo emergence of vaccine escape mutants and not in their subsequent spread, we model the disease dynamics in the absence of the vaccine escape variant. From the number of wild-type infections, we can estimate the risk of vaccine escape evolution. The flow diagram of the model is shown in figure 1*a*.

Once vaccines become available, there is a limited but constant supply of vaccine doses that allows injections to be administered at total rate *λ*. We assume that vaccine doses are only given to individuals who have never been infected by the virus (i.e. infected and recovered individuals do not receive any (further) vaccination). Up to undetected and early cases, this assumption is in line with the recommendations by the Robert Koch Institute in the early stages of vaccine availability [18]. At later stages, it constitutes a simplification. Any vaccine dose can either be used as a first or as a second shot. If the time interval between the two injections is chosen as *ω* and the second shots are administered at rate (1/*ω*)*V*_*H*_ (where *V*_*H*_ is the fraction of susceptible half-vaccinated individuals), the rate of first dose injections is limited to *λ* − (1/*ω*)*V*_*H*_. The time interval *ω* determines the vaccination strategy: it can either be set to the interval *ω*_min_ recommended by the manufacturer or be stretched to increase the rate at which unvaccinated individuals receive the first dose. We set the maximal interval as *ω*_max_ = 5 · *ω*_min_. In either case, by construction, there are always sufficient vaccine doses available to administer second doses as scheduled (see appendix A). This modelling choice is in line with real-life vaccine stock management in which sufficient doses are usually withheld for second shots. For simplicity, we assume that the efficacy of the first dose remains constant over time and that the dosing interval *ω* has no effect on the efficacy of the second dose.

Unvaccinated and partially vaccinated individuals can become infected with the virus. Vaccination with both doses, however, entirely blocks infection with the wild-type strain (which is a simplifying assumption; e.g. Dagan *et al.* [19]). The transmission coefficient between unvaccinated infected individuals and unvaccinated susceptible individuals is given by *β*. Vaccination with the first dose follows a leaky mode of action: it reduces susceptibility by a factor 1 − *x* and transmissibility by a factor 1 − *y* [6,20–22]. The parameter *x* is thus the ‘efficacy for susceptibility’ and the parameter *y* the ‘efficacy for transmissibility’ of a single dose. We assume that infected individuals recover at rate *γ*, irrespective of their vaccination status, i.e. the time-course of infectiousness does not change with vaccination with a single vaccine dose. Upon recovery, all infected individuals gain full immunity over the relevant time scales of the pandemic. We assume that vaccine escape variants against which the vaccine has reduced efficacy evolve and become dominant within unvaccinated individuals at *per capita* rate *μ*_*I*_ and within half-vaccinated individuals at *per capita* rate *μ*_*V*_. We mostly focus on the case *μ*_*V*_ ≥ *μ*_*I*_, but also briefly consider *μ*_*V*_ < *μ*_*I*_. Once the vaccine escape variant is dominant in one individual, it can spread across the host population. However, here we focus on the emergence of these strains and not on their future dynamics.

Before the escape variant arises, the dynamics of the epidemic is described by the differential equations

2.1 $$\begin{array}{rl} \frac{dS}{dt} & =-\left(\lambda -\frac{1}{\omega }V_{H}\right)-\beta S(I+(1-y)V_{H,I}),\\ \frac{dV_{H}}{dt} & =\left(\lambda -\frac{1}{\omega }V_{H}\right)-\frac{1}{\omega }V_{H}-(1-x)\beta V_{H}(I+(1-y)V_{H,I}),\\ \frac{dV_{F}}{dt} & =\frac{1}{\omega }V_{H},\\ \frac{dI}{dt} & =\beta S(I+(1-y)V_{H,I})-\gamma I,\\ \frac{dV_{H,I}}{dt} & =(1-x)\beta V_{H}(I+(1-y)V_{H,I})-\gamma V_{H,I}\\ \mathrm{and}\;\;\frac{dR}{dt} & =\gamma I+\gamma V_{H,I}, \end{array}\}$$

where the first three equations describe the fraction of individuals that are uninfected and either unvaccinated (*S*), half-vaccinated (*V*_*H*_) or fully vaccinated (*V*_*F*_). The next two equations describe individuals that are infected and either unvaccinated (*I*) or half-vaccinated (*V*_*H*,*I*_). The last equation describes all individuals that are immune due to naturally acquired immunity following an infection (*R*). In the electronic supplementary material, we study a version of our model that includes an incubation period modelled by an exposed class *E* (SEIR model), which is more realistic for COVID (electronic supplementary material, S1 and figure S1).

Once every individual has received the first dose (or has been infected by the virus), i.e. *S* = 0, there is no reason anymore to delay the second dose. However, it is possible that in a delay strategy, many half-vaccinated individuals are still waiting for the second shot and that the available doses are still not sufficient to administer the second doses at rate 1/*ω*_min_. Therefore, once *S* = 0, the second shot is administered at a rate given by the minimum of (1/*ω*_min_)*V*_*H*_ and *λ*. (As long as *S* > 0, there are always enough doses available to vaccinate at a steady *per capita* rate 1/*ω*, see appendix A.) Ultimately, every individual in the population has either been vaccinated or has acquired immunity through infection.

We choose the model parameters in accordance with values for the COVID pandemic. We set the infectious period to 5 days, i.e. 1/*γ* = 5 days (cf. [11]) and the minimal interval between the vaccine doses to *ω*_min_ = 20 days, which is the recommended interval for the Pfizer–BioNTech vaccine. For the rate of vaccine roll-out, if not stated otherwise, we choose λ=0.2%, which roughly corresponds to the rate in Germany during March 2021 [23].

As a default, we assume the reproductive number in the absence of any immune individuals to be *R*_*C*_ = *β*/*γ* = 1.2, which is much smaller than the basic reproductive number of SARS-CoV-2 in the absence of control measures [24]. Our choice means that there are control measures in place, but they are insufficient to control the spread of the disease. There are no estimates for the per-patient mutation rates *μ*_*I*_ and *μ*_*V*_. The absolute values only change the number of new mutant infections, while the qualitative results in our model depend on the ratio *μ*_*V*_/*μ*_*I*_ (see below for details). To account for the uncertainty in this ratio, we consider *μ*_*V*_/*μ*_*I*_ ∈ {0.1, 1, 10}. We set *μ*_*I*_ = 10^−6^. Our primary focus is a scenario in which the vaccination campaign starts several months into the pandemic when a noticeable fraction of the population has already been affected by the virus. We define *t* = 0 as the start of the vaccination campaign. As initial conditions, we set *I*(0) = 2.4 × 10^−3^ and *R*(0) = 4.2 × 10^−2^, which corresponds respectively to twice the sum of the reported number of new cases in the 5 days before 1 January 2021 in Germany and twice the reported number of total cases on 1 January 2021 [25], making the assumption that only 50% of the cases are detected [26]. The number of recovered cases is in line with seroprevalence studies in Germany at this time [27,28]. To account for the uncertainty in the initial value of infectious cases *I*(0), we explore the dynamics with other values in the electronic supplementary material, finding that the qualitative results remain the same (figure S8).

We numerically integrate the differential equations using Python; see our Jupyter notebook that is available in the electronic supplementary material. Example dynamics are shown in figure 1*b*–*d*. Figure 1*b* shows the dynamics in the absence of vaccination. In figure 1*c*, the second dose is given as soon as possible (*ω* = *ω*_min_), while figure 1*d* shows the dynamics under the maximal delay strategy (*ω* = *ω*_max_).

We aim to determine how the strategy affects the number of (severe) cases and the risk of vaccine escape. The first quantity that we consider is the normalized cumulative number of severe cases from the start of the vaccination campaign until the end of the pandemic, provided no vaccine escape mutants evolve,

2.2 $$B=\int_{0}^{\infty }\gamma I(t)+(1-z)\gamma V_{H,I}(t)\;dt,$$

where *z* is the relative efficacy against severe disease of a single dose of the vaccine. We refer to *B* as the burden. Without loss of generality, we have normalized the fraction of severe cases by the probability that an unvaccinated individual is a severe case. The total burden can be decomposed into the burden $B_{S}=\int_{0}^{\infty }\gamma I(t)\;dt$ arising from unvaccinated individuals and the burden $B_{H}=\int_{0}^{\infty }(1-z)\gamma V_{H,I}(t)\;dt$ arising from half-vaccinated individuals. In the main text, we will restrict our analysis to *z* = 0, i.e. a single dose does not reduce disease severity (but see electronic supplementary material, figure S4). In this case, the burden describes the cumulative fraction of infected individuals since the onset of vaccination (*B*(*z* = 0) = *R*(∞) − *R*(0)).

The second quantity of interest is the escape risk defined as the cumulative fraction of patients in whom vaccine escape mutants evolve after vaccine roll-out has started in the population (assuming that none are spreading yet at that time):

2.3 $$M=\int_{0}^{\infty }\mu_{I}I(t)+\mu_{V}V_{H,I}(t)\;dt.$$

As for the burden, it can be insightful to decompose the total escape risk *M* according to the vaccination status of the patients into $M_{S}=\int_{0}^{\infty }\mu_{I}I(t)\;dt$ and $M_{H}=\int_{0}^{\infty }\mu_{V}V_{H,I}(t)dt$. The measure *M* does not account for differences in onward transmission of the vaccine escape variant from unvaccinated and half-vaccinated individuals in whom it has newly evolved. Even if the escape variant becomes dominant within a host, viral loads may well be different in these two groups of patients due to the different within-host dynamics, which may lead to differences in transmission. In the main text, we avoid making assumptions on the degrees of onward transmission (note that the efficacies *x* and *y* for the wild-type variant may not apply to newly evolved mutants). In the electronic supplementary material, we include the first step of onward transmission of new vaccine escape variants to the next host into the measure *M*, assuming that a single vaccine dose also reduces the transmissibility of the mutant variant; see electronic supplementary material, figures S5, S6 and S7.

In a non-deterministic world, vaccine escape mutants may or may not evolve. The sum *μ*_*I*_ *I*(*t*) + *μ*_*V*_ *V*_*H*_(*t*) can also be interpreted as a stochastic rate, and the integral quantifies the total risk over the course of the pandemic (not taking into account the risk prior to the vaccine roll-out, which is independent of the chosen vaccination strategy). More precisely, the probability that vaccine escape mutants evolve is given by

2.4 $$P_{\mathrm{escape}}=1-e^{-NM},$$

where *N* is the total population size (similarly, the probability that vaccine escape has already evolved prior to the vaccine roll-out can be estimated as $1-e^{-N\mu_{I}R(0)/\gamma }$). It should be noted that this is the probability of their mere appearance. That does not mean that they will spread. For example, some of those patients in whom vaccine escape mutants evolve may not infect anyone, in which case the mutation is lost again from the population. For shortness, we often refer to *M* as the risk of vaccine escape in the following, but it should be kept in mind that the probability of vaccine escape is not directly given by *M* but by equation (2.4) and that this does not involve any probability of establishment of the vaccine escape mutant in the population.

## 3. Results

### 3.1. When can conflicts between reducing the burden and the escape risk appear?

What is the relationship between the burden *B* (with *z* = 0) and the fraction of new mutant infections *M*? The fraction of mutants can be rewritten as $M=\mu_{I}\int I+(\mu_{V}/\mu_{I})V_{H,I}\;dt$. Thus, the qualitative effect of increasing or decreasing *ω* only depends on the ratio *μ*_*V*_/*μ*_*I*_, but not on their individual values. Comparing to the total burden $B=\gamma \int I+V_{H,I}\;dt$, we see that there is no conflict between minimizing both quantities if *μ*_*V*_ = *μ*_*I*_, since in that case both quantities are proportional to each other, *M* ∝ *B*. However, if *μ*_*V*_ > *μ*_*I*_, the two quantities are no longer proportional to each other, and it is conceivable that *M* increases with a change in *ω*, while *B* decreases. Similarly, if *μ*_*V*_ < *μ*_*I*_, a delay strategy may decrease *M* but increase *B*. By contrast, we always have *M*_*S*_ ∝ *B*_*S*_ and *M*_*H*_ ∝ *B*_*H*_, i.e. within the two sub-populations of susceptibles and half-vaccinated patients there is no conflict and a reduction in burden always implies a reduction in escape risk.

### 3.2. How do efficacies for either susceptibility or transmissibility affect the burden and the risk of vaccine escape?

**Figure 2.**
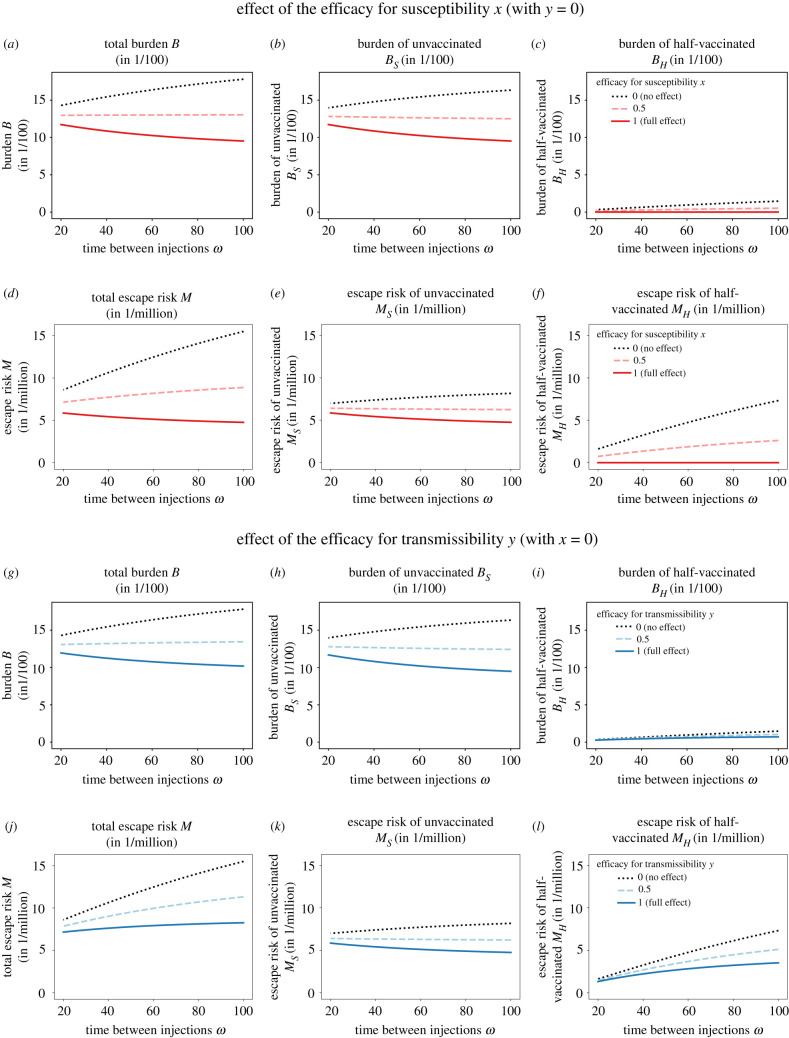
Burden and number of new mutant infections (escape risk) as a function of the interval between the two doses for the two limiting cases, in which the first dose has an effect on (*a*–*f*) susceptibility only (*y* = 0) or on (*g*–*l*) transmissibility only (*x* = 0). For illustrative purposes, we show the differences in burden per hundred people Δ*B* × 10^2^ and the differences in escape risk per million people Δ*M* × 10^6^. The black dotted lines, where the first dose has no effect at all (*x* = *y* = 0), are identical in the two parts of the figure. Reductions in susceptibility and transmissibility both reduce the burden and the risk of vaccine escape. The consequences of a delay of the second shot depend on the effects of the first dose. A delay may increase both the burden and the risk of vaccine escape (dotted black line in *a* and *d*/*g* and *j*), decrease both quantities (solid red line in *a* and *d*), or decrease the burden at the cost of an increased risk of vaccine escape (solid blue line in *g* and *j*). The figure shows results for our default parameter set with the ratio of mutation rates *μ*_*V*_/*μ*_*I*_ = 10.

**Figure 3.**
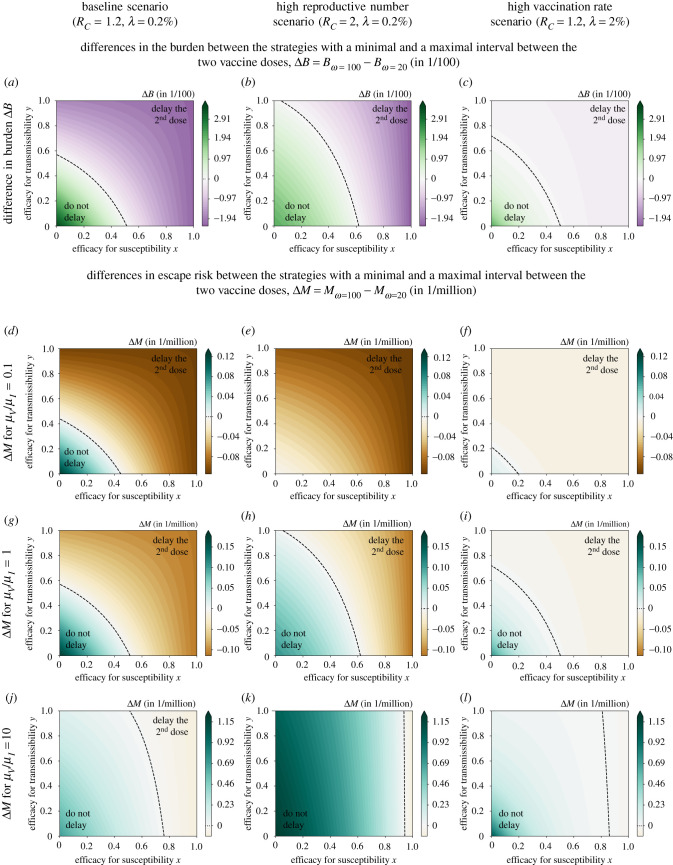
Differences in the burden and the number of new mutant infections (escape risk) between the strategies with a minimal and a maximal interval between the two vaccine doses, Δ*B* = *B*_*ω*=100_ − *B*_*ω*=20_ and Δ*M* = *M*_*ω*=100_ − *M*_*ω*=20_, depending on the efficacies for susceptibility *x* and transmissibility *y* elicited by the first dose. If the difference is positive, a delay increases the burden/escape risk (do not delay). If it is negative, a delay reduces the burden/escape risk (delay the second dose). For illustrative purposes, we show the differences in burden per hundred people Δ*B* × 10^2^ and the differences in escape risk per million people Δ*M* × 10^6^. The three columns correspond to our baseline scenario with the default parameter set (left column), a high reproductive number scenario (middle column), and a scenario with a high vaccination rate *λ* (right column). We consider three different ratios of the *per capita* mutation rates in unvaccinated and half-vaccinated individuals. As expected, for *μ*_*V*_/*μ*_*I*_ = 1 (*g*–*i*), the same strategy minimizes both the burden and the risk of vaccine escape, irrespective of *x* and *y*. For *μ*_*V*_ > *μ*_*I*_ (*j*–*l*), there is a parameter range, in which a delay reduces the burden but increases the risk of vaccine escape. For *μ*_*V*_ < *μ*_*I*_ (*d*–*f*), by contrast, a delay increases the burden but decreases the escape risk in some parameter regime. Note that the colour scale is the same across rows but not across columns.

Before exploring the entire range of possible effect sizes *x* and *y* of the first vaccine dose, we consider the two limiting cases, in which the first vaccine dose has an effect either on susceptibility only (figure 2*a*–*f*) or on transmissibility only (figure 2*g*–*l*), but not on both simultaneously, and consider *μ*_*V*_/*μ*_*I*_ = 10. For our default parameter set, reductions in susceptibility and transmissibility reduce both the burden and the escape risk, and they can both change the effect of the delay between the injections. With respect to the total burden, efficacies for susceptibility and transmissibility have overall very similar effects, both quantitatively and with respect to their influence on the optimal strategy (compare figure 2*a* and figure 2*g*, but see results for higher *R*_*C*_ in figure 3, where this does not hold). By contrast, for the appearance of vaccine escape variants, it makes a difference whether the first dose reduces susceptibility or transmissibilty (compare figure 2*d* and figure 2*j*). The discrepancy comes from slight differences in the fraction of half-vaccinated patients that do not affect the total burden much but amplify in *M*_*H*_ (see especially the solid lines in figure 2*f*,*l*).

In both cases, the burden mainly stems from unvaccinated individuals that become infected (compare *B*_*S*_ with *B*_*H*_ in figure 2). The interval between the two doses has not only a direct effect on this burden by affecting the fraction of unvaccinated individuals but also an indirect effect by changing the transmission dynamics (e.g. for *x* = 0, *B*_*S*_ increases with *ω*, although a longer delay reduces the fraction of unvaccinated susceptible individuals *S*). In a similar way, if the first dose has no effect on transmissibility (*y* = 0), most mutants emerge from unvaccinated individuals. By contrast, if the first dose has no effect on susceptibility (*x* = 0), evolution within half-vaccinated individuals substantially contributes to the emergence of vaccine escape even if the first dose blocks transmission of the wild-type virus completely (*y* = 1; see figure 2*l*).

Regarding the effect of a potential delay of the second shot, we observe that the strategy *ω* that minimizes the burden is always either the no-delay strategy (*ω* = *ω*_min_) or a maximal delay (*ω* = *ω*_max_), but never an intermediate interval between the two doses (figure 2*a*,*g*). The same holds true for the fraction of new mutant infections (figure 2*d*,*j*). If the first vaccination has neither an effect on susceptibility nor on transmissibility, both the burden and the escape risk increase with the time between the two injections. For strong effects in either susceptibility or transmissibility, an increase in the delay between injections reduces the burden (again, this is not true for higher *R*_*C*_; see below). For the escape risk, the picture is different. With a strong efficacy for susceptibility, a delay of the second shot reduces the escape risk. In that case, the effects of a delay on the burden and on the vaccine risk align. By contrast, if the vaccine has no effect on susceptibility (*x* = 0), delaying the second dose increases the risk of vaccine escape, irrespective of how well the first dose blocks onward transmission of the wild-type virus (*y* = 1; solid curve in figure 2*j*). The effects on the burden and on the risk of vaccine escape diverge in this case.

We therefore can conclude from these limiting cases that conflicts between reducing the burden and the risk of vaccine escape can exist (see *x* = 0, *y* = 1), but the reduction in cases can also outweigh the increased risk of within-host evolution of vaccine escape (see *y* = 0, *x* = 1).

### 3.3. In which range of efficacies for both transmissibility and susceptibility do trade-offs emerge?

**Figure 4.**
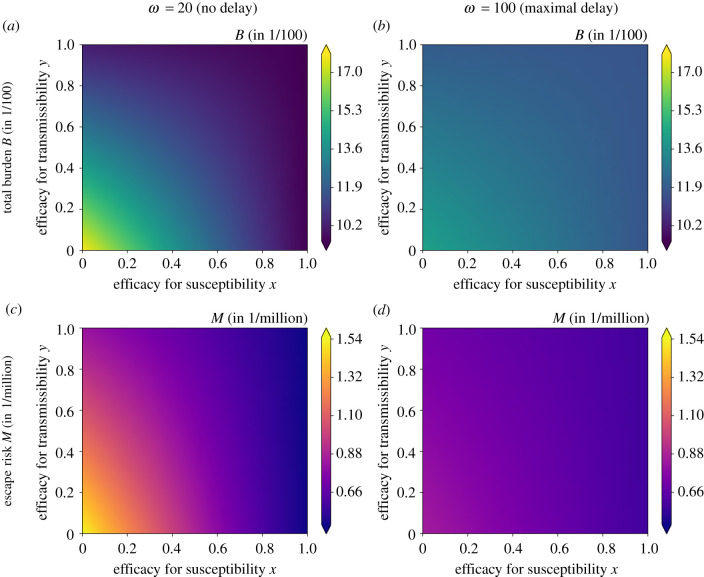
Total burden *B* and total number of new mutant infections *M* for a minimal interval between the two doses (*a*,*c*) and for a maximal delay (*b*,*d*), depending on the efficacies for susceptibility *x* and transmissibility *y* elicited by the first dose. For illustrative purposes, we show the differences in burden per hundred people Δ*B* × 10^2^ and the differences in escape risk per million people Δ*M* × 10^6^. Increasing the efficacy for susceptibility or transmissibility reduces the total burden and the escape risk, but in quantitatively different ways such that a trade-off between them regarding the optimal time between injections *ω* can emerge. The figure shows results for our default parameter set with the ratio of mutation rates *μ*_*V*_/*μ*_*I*_ = 10.

To investigate more closely under which circumstances the effect of the strategy on the burden and the fraction of escape mutants diverge, we proceed to explore the entire range of efficacies for susceptibility and transmissibility and vary other parameters as well. As for the limiting cases, we found that the optimal strategy is either no delay or a maximal delay. We therefore focus on the burden and the escape risk with the recommended interval of $\omega_{\min}=20\;\mathrm{days}$ and a maximally stretched interval of *ω*_max_ = 100 days. To determine which strategy is optimal under the respective criterion, we consider the differences Δ*B* = *B*_*ω*=100_ − *B*_*ω*=20_ and Δ*M* = *M*_*ω*=100_ − *M*_*ω*=20_ and ask when they are larger than zero (do not delay) or smaller than zero (delay the second dose); see figure 3. For reference, i.e. to put the differences in Δ*B* and Δ*M* into context of absolute values of *B* and *M*, the absolute burden and fraction of new mutant infections with our default parameter set is given in figure 4 for both values of *ω*.

We already know from the analysis of the limiting cases that neither strategy is optimal in minimizing the burden across the entire range of *x* and *y*. This can be further seen in figure 3*a*–*c*. If the effect of the first dose is sufficiently strong, the delay strategy is favoured. If it is weak, the second dose should not be delayed. The area in which delaying the second dose is beneficial is reduced if the reproductive number *R*_*C*_ is higher, i.e. if the infection rate in the population is larger (cf. figure 3*a* and figure 3*b*). In that case, a reduction of the transmissibility on its own without any effect of the first dose on susceptibility is insufficient to justify a delay strategy. If more vaccine doses are available (higher vaccination rate *λ*; figure 3*c*), the choice of the strategy becomes less important, with differences in outcomes being smaller.

Both strategies affect the burden and the fraction of escape mutants equivalently if *μ*_*V*_ = *μ*_*I*_ (compare the first and third rows of the figure). For *μ*_*V*_ > *μ*_*I*_, as expected from the general considerations above, the range of *x* and *y* favouring a delay strategy is larger for the burden than for the number of mutants (figure 3*j*–*l*). In that case, a parameter range opens up in which a delay strategy reduces the burden but increases the fraction of new mutant infections. By contrast, if *μ*_*V*_ < *μ*_*I*_ (figure 3*d*–*f*), a delay strategy may not be optimal for reducing the burden, but reduce the risk of vaccine escape. In the electronic supplementary material, we show that the results are not qualitatively different for the SEIR version of our model (electronic supplementary material, figures S2 and S3). We furthermore study in the electronic supplementary material how the trade-off changes if the first dose reduces the disease severity (*z* > 0). In this case, trade-offs also occur for *μ*_*V*_/*μ*_*I*_ = 1. For *μ*_*V*_/*μ*_*I*_ = 10, the parameter range in which a trade-off occurs is even larger than in the absence of an effect of the first dose on disease severity (i.e. for *z* = 0), while it gets smaller if *μ*_*V*_/*μ*_*I*_ = 0.1 (electronic supplementary material, figure S4). Reduced transmissibility of the mutant variant in half-vaccinated individuals—if taken into account in the assessment of the vaccine escape risk by including the first step of onward transmission—reduces the critical area in the *x*–*y* plane, in which we observe trade-offs, for *μ*_*V*_ > *μ*_*I*_ (electronic supplementary material, figures S6 and S7).

The differences Δ*B* and Δ*M* between the two strategies can be substantial, such that they become relevant in choosing vaccination strategies. If vaccine doses are scarce, the strategy can change the infected fraction of the population by several percentage points of the population for some combinations of *x* and *y*. To put this into perspective, the cumulative fraction of COVID cases in Germany in April 2021 (four months after the start of the vaccination campaign) was around 7% [25,27] (assuming as above that about 50% of all cases are detected [26]). Hence, the effect of the strategy is in some parameter regions of the same order of magnitude as the cumulative number of cases in Germany at the time this paper was written. Likewise, the strategy can substantially influence the total fraction of new mutant infections, doubling *M* in some cases and with it increasing the risk that vaccine escape mutants evolve.

### 3.4. What changes if vaccines are available right from the start of the pandemic?

**Figure 5.**
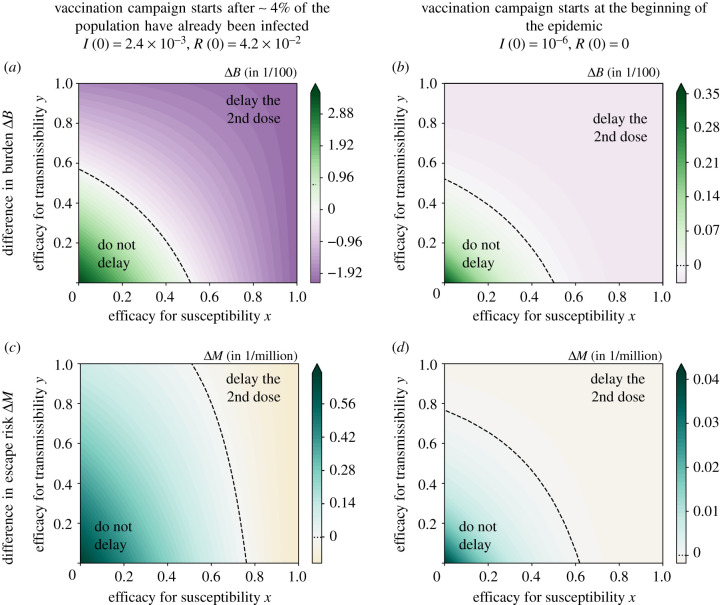
Comparison between a scenario in which vaccines become available during a pandemic (*a*,*c*) and a scenario where they are available right from the start (*b*,*d*). The figure shows the differences in the burden and in the number of new mutants infections between the strategies with a minimal and a maximal interval between the two vaccine doses, Δ*B* = *B*_*ω*=100_ − *B*_*ω*=20_ and Δ*M* = *M*_*ω*=100_ − *M*_*ω*=20_. For illustrative purposes, we show the differences in burden per hundred people Δ*B* × 10^2^ and the differences in escape risk per million people Δ*M* × 10^6^. When vaccines are available from the start, differences between strategies are much smaller. (Note the different scales of the colour gradients between columns and between this figure and figure 3.) The figure shows results for our default parameter set with the ratio of mutation rates *μ*_*V*_/*μ*_*I*_ = 10.

We finally compare the results to a scenario in which the vaccine is available right from the start of the pandemic, for which we choose *I*(0) = 10^−6^ and *R*(0) = 0 (figure 5). Such a scenario may be relevant in case escape mutations necessitate a novel vaccination campaign and that the vaccine is available before the new variant is present in all countries. In this case, the parameter range in which a delay strategy minimizes the risk of escape mutants is enlarged (compare figure 5*c* and figure 5*d*). However, the benefit of either strategy in reducing the burden or the risk of vaccine escape is much smaller than for our primary scenario, in which a vaccine becomes available only during the pandemic (compare the two columns).

## 4. Discussion

When vaccines are scarce, should we increase the timing between the two doses? Using an SIR model, extended by half-vaccinated (first dose only) and fully vaccinated (two doses) individuals, we explored when the choice of the interval between the two doses—delay or no delay—leads to conflicts between reducing the burden and the risk of vaccine escape evolution. On short time scales, reducing the burden is the primary goal, but the risk of vaccine escape could pose a major societal problem on a longer time scale.

### 4.1. What does the SIR model find?

If the first dose has only a weak effect, a delay of the second dose increases both the burden and the fraction of new mutant infections. If the first dose has a sufficiently strong effect, a delay is beneficial in both respects. However, this latter area in the susceptibility–transmissibility efficacy (*x*–*y*) plane is small and limited to an extremely high efficacy for susceptibility if the within-host evolution of vaccine escape is much larger in half-vaccinated than in unvaccinated individuals. Between these two parameter ranges in which both criteria suggest the same interval between the two doses, there is a parameter region in which a delay reduces the burden—but at the cost of an increased risk of vaccine escape. This region is absent if vaccine escape evolution is equally likely within half-vaccinated and unvaccinated patients (unless a single vaccine dose reduces the risk of severe disease as considered in electronic supplementary material, S2). If vaccination with the first dose reduces the *per capita* rate of vaccine escape, a delay strategy may reduce the risk of vaccine escape, but increase the burden. These results also hold true if an exposed class is included into the model. If vaccine escape is more likely in half-vaccinated than in unvaccinated individuals, the critical parameter region is even larger if the first dose reduces the risk of severe disease. It becomes smaller if onward transmission of the new mutant to the next host is reduced by the first drug dose but does not even vanish if the reduction is as strong as for the wild-type variant of the virus.

Our model suggests that the conditions under which a delay strategy is favourable for reducing the burden are more restrictive with a higher reproductive number *R*_*C*_. This is the opposite of what has been found by Matrajt *et al.* [6], comparing a strict one-dose to a two-dose strategy. There are several differences between the models. For example, Matrajt *et al.* [6] assume that all individuals are vaccinated at the same time but immunity takes time to build up following vaccination, while we assume that vaccine roll-out is a continuous process over time but once an individual has received a vaccine shot, the effect is immediate. Other differences include incomplete versus complete vaccine coverage, the presence and absence of asymptomatic infections, and incomplete versus complete protection with two doses.

### 4.2. What affects the fate of new escape variants?

We only consider the emergence of vaccine escape mutants but do not track their spread (except for a single transmission step, which we consider in the electronic supplementary material, S3). As already mentioned in the model section, the fate of these mutants is subject to stochasticity as long as they are rare [29]. Especially when the distribution of secondary cases is overdispersed—i.e. when a small number of patients infect many others, while the majority of patients infect only few or no other individuals—it is likely that the mutation is lost again from the population [30]. The heterogeneity in transmission has been estimated to be rather high for SARS-CoV-2 [31,32]. Beyond these stochastic effects, there are many factors that affect the spread of escape variants by changing their reproductive number, and we only discuss a few examples here. Social distancing, contact tracing, and isolation of infecteds control not only the wild-type virus but also escape variants. For our model, we assumed *R*_*C*_ to be constant throughout the pandemic, but in reality the transmission coefficient changes over time due to control measures, changes in behaviour, and also up to some extent seasonality. This includes in particular the extent at which social distancing restrictions are lifted already during a vaccination campaign. This affects the establishment of escape variants [29], but also their propagation once frequent. A further factor that is crucial for the fate of escape mutants, short term and long term, is the degree up to which vaccination is still effective against them. Cobey *et al.* [16] argue that vaccination will likely still grant some level of protection against escape variants. This means that they would not spread well in a population with high vaccination coverage. Yet, escape mutants may accumulate additional mutations over time that increase their degree of vaccine resistance. Apart from residual vaccine protection to escape mutants, if different vaccines using different antigenic targets or variants of the same target are employed, the spread of mutants across the population escaping from one vaccine is likely still hampered by the other vaccines [33]. Spread of escape variants is moreover affected by the protection provided by naturally acquired immunity, which may not always be sufficient to prevent reinfection [34].

### 4.3. Are effects on the burden and on vaccine escape equally predictable?

The difference that the choice of strategy makes can be substantial. Whether an extended interval between shots increases or decreases the burden crucially depends on the efficacies for susceptibility and transmissibility elicited by the first dose. The number of studies estimating the effects of the first (and second) doses of the various COVID vaccines is currently (i.e. in spring 2021) rapidly growing. With sufficient information, we can probably be rather confident about the consequences of our choice with respect to the disease burden. Matters are much more complicated when it comes to assessing the risk of vaccine escape, which requires one to make predictions about evolution in a highly complex and dynamic environment. In our simple model, the range of first dose effects for which a reduction in burden comes at an increased risk of vaccine escape depends on the relative probabilities of within-host evolution of escape mutants in half-vaccinated and unvaccinated individuals. There is concern that vaccine escape mutants evolve more easily in partially immune individuals, but we do not know whether this is really the case and, if so, how much more likely it is, or if vaccine escape is not even less likely in half-vaccinated individuals [11,13,16,17]. We generally do not know how easily the virus can escape from immunity nor do we know up to which degree mutants will evade vaccine-induced immunity and potentially also naturally acquired immunity nor what their degree of cross resistance to other vaccines is going to be. And even if we knew all this, the appearance and establishment of vaccine escape mutants would still be a probabilistic event that may or may not happen. Moreover, escape mutants can also be imported from other regions [35]. When sufficient information on the effectiveness of the first dose and the duration of immunity is known, it is hence weighing an immediate assessable benefit against an unknown future risk with unknown consequences.

### 4.4. Conclusion

Our model is not suitable to solve the dilemma nor to make any concrete recommendations. It only contains the most essential elements necessary to describe the epidemiological dynamics, which, of course, requires making many simplifying assumptions. The *per capita* rates of escape evolution are model parameters, and the model explores the population-level consequences, given a certain ratio between the rates in half-vaccinated and unvaccinated patients. Such a fundamental model shows in a transparent manner how the verbal arguments that have been brought forward play out in a quantitative model. Maybe most importantly, the model allows one to see that a delay strategy can increase or decrease the risk of vaccine escape and to identify when conflicts between reducing the burden and the risk of vaccine escape arise, where the effects of the first dose in terms of efficacies for susceptibility and transmissibility and the relative risks of vaccine escape in half-vaccinated and unvaccinated patients are key parameters. It could also provide a starting point for more detailed models that take further complications into account. Relevant extensions include the implementation of a risk structure with possibly different strategies across risk groups, heterogeneity in vaccine efficacy among individuals, increasing vaccine availability over time, and a more realistic dynamics of the pandemic, where the reproductive number fluctuates with tightening and loosening of control measures and seasonality. Maybe most importantly, future models also need to address the influence of the vaccination strategy on the spread of escape variants across the host population. Our current model clearly shows that the decision to delay the second dose can influence the future number of cases and the risk of the evolution of escape variants. It furthermore shows that knowledge about both the efficacy for susceptibility and the efficacy for transmissibility is needed to make a quantitatively informed decision. We hope that our model helps to provide a more solid foundation for the discussion on vaccination strategies.

## Data Availability

The Jupyter notebook for running the model and reproducing the figures is available as electronic supplementary material [36].

## Appendix A. Availability of second doses

In this appendix, we show that there are at all times enough doses available to administer the second dose after the chosen time interval *ω*, as long as *S*(*t*) > 0, i.e. there never arises a situation in which the second dose needs to be further delayed due to vaccine shortage. For this, we need to show that *λ* > (1/*ω*)*V*_*H*_.

We first consider a disease-free population (*I*(*t*) = *V*_*H*,*I*_(*t*) = 0 for all *t*). We denote the fraction of half-vaccinated individuals in this scenario by *V*_*H*,0_. From equation (2.1), their dynamics is given by

A 1 $$\frac{dV_{H,0}}{dt}=\left(\lambda -\frac{1}{\omega }V_{H,0}\right)-\frac{1}{\omega }V_{H,0}\;\mathrm{with}\;V_{H,0}(0)=0,$$

which solves to

A 2 $$V_{H,0}(t)=\frac{\lambda \omega }{2}(1-e^{-(2/\omega )t}).$$

With this, we have

A 3 $$\lambda -\frac{1}{\omega }V_{H,0}=\lambda \left(\frac{1}{2}+e^{-(2/\omega )t}\right)>0.$$

We now turn to a population in which the virus is spreading. In this case, as given in equation (2.1), there is an infection in the differential equation describing the changes in the fraction of half-vaccinated individuals:

A 4 $$\begin{array}{rl} & \frac{dV_{H}}{dt}=\left(\lambda -\frac{1}{\omega }V_{H}\right)-\frac{1}{\omega }V_{H}\\ & \;\;-\underset{\equiv V_{H}\;f(t)}{\underbrace{\left(1-x)\beta V_{H}(I+(1-y)V_{H,I}\right)}}\\ & \;\mathrm{with}\;V_{H}(0)=0. \end{array}$$

Comparing to equation (A 1), we see that

$$\frac{dV_{H}}{dt}\leq \frac{dV_{H,0}}{dt}\;\mathrm{and}\;V_{H}(0)=V_{H,0}(0)=0.$$

Therefore,

$$V_{H}(t)\leq V_{H,0}(t)\Rightarrow \lambda -\frac{1}{\omega }V_{H}(t)\geq \lambda -\frac{1}{\omega }V_{H,0}(t)>0,$$

where the last step follows from equation (A 3). This implies *λ* > (1/*ω*)*V*_*H*_.
